# Adoption of Artificial Intelligence (AI)-Based Computerized Tomography (CT) Evaluation of Comprehensive Nursing in the Operation Room in Laparoscopy-Guided Radical Surgery of Colon Cancer

**DOI:** 10.1155/2022/2180788

**Published:** 2022-03-08

**Authors:** Xiuping Duan, Dechun Su, Haiwei Yu, Wei Xin, Yang Wang, Ziming Zhao

**Affiliations:** ^1^Operating Room, Affiliated Hongqi Hospital of Mudanjiang Medical University, Mudanjiang 157011, Heilongjiang, China; ^2^Department of Obstetrics, Affiliated Hongqi Hospital of Mudanjiang Medical University, Mudanjiang 157011, Heilongjiang, China; ^3^Department of Emergency, Affiliated Hongqi Hospital of Mudanjiang Medical University, Mudanjiang 157011, Heilongjiang, China

## Abstract

This research aimed to discuss the application of traditional nonlocal mean (NLM) algorithm-based computerized tomography (CT) images in intervention evaluation of the nursing for patients performing laparoscopy-guided radical surgery of colon cancer. A total of 100 patients who were diagnosed with colon cancer after enteroscopy and performed laparoscopic radical surgery were chosen as the research objects. They were divided into an observation group (comprehensive nursing in operation room) and a control group (routine nursing), each of which included 50 cases. All cases received CT examination. Meanwhile, the improved traditional NLM (INLM) algorithm was proposed, and the effects of image reconstruction were analyzed to improve the quality of CT images. The result showed that structural similarity index measure (SSIM) and figure of merit (FOM) of INLM were obviously higher than those of filtered back projection (FBP) algorithm and NLM algorithm, and the average running time was significantly less than that of FBP algorithm and NLM algorithm (*P* < 0.05). The operation time and the amount of intraoperative blood loss of patients in the observation group were both less than those of patients in the control group, and differences had statistical significance (*P* < 0.05). Besides, the time of getting out of bed, ventilation recovery time, postoperative meal time, stomach tube encumbrance time, and catheter encumbrance time of patients in the observation group were all less than those of patients in the control group, and the differences had statistical significance (*P* < 0.05). In the observation group, there were 3 cases with postoperative complications, and 2 out of them got incision infection while 1 suffered from constipation. In contrast, there were 9 cases with postoperative complications in the control group, 3 of which were patients with incision infection, and 2 suffered from urinary retention while the other 4 suffered from constipation. According to the above results, the INLM algorithm proposed in this research could improve the image reconstruction accuracy of traditional algorithm, shorten the running time, and enhance the overall diagnostic efficiency. The comprehensive nursing in operation room with laparoscopic radical surgery of colon cancer could improve the cure rate and prognosis of patients, so it was worthy of clinical promotion and application.

## 1. Introduction

Colon cancer is a kind of common malignant tumor occurring in colon parts of digestive tract, and it is the third commonest tumor among all gastrointestinal cancers [1, 2]. The clinical manifestations of colon cancer vary from the size and position of its lesions to the pathological type [3–5]. However, there is no clinical symptom among many patients with colon cancer at early stage, but a series of common symptoms related to colon cancer emerged as the disease develops and lesions expand constantly, such as the increasing number of defecations, defecation with blood, stomachache, diarrhea or constipation, ileus, malaise, loss of weight, and anemia [6, 7]. The treatment of colon cancer is emphasized firstly on surgical incision and on the combined preoperative chemotherapy and radiotherapy to increase the surgical incision rate, to reduce the postoperative recurrence rate, and to enhance the survival rate [8]. With the improvement of laparoscopic technology and the update of medical equipment, laparoscopy-guided radical surgery can be adopted not only in benign lesions of stomach and stomach cancer at early stage, but also in the treatment of advanced gastric cancer gradually [9].

In the general clinical treatment, colon cancer is evaluated by barium meal in digestive tract, barium double contrast agent enema X-ray, and fibroendoscopy. These methods can play a significant role in localization and characterization of lesions, but only the lesions in lumen can be observed. Gastrointestinal wall infiltration, the relationship between tumors, and adjacent tissues cannot be shown clearly [10–12]. Magnetic resonance imaging (MRI) can examine pelvic cavity from different angles and display colon cancer ideally. Besides, the invasion of mucosa and submucosa by tumors can be observed using small field and internal rectal coil [13]. To some extent, the diagnosis of colon cancer by CT imaging examination is valuable because it can detect small and hidden lesions in colon and rectum and evaluate the relationship between cancer and peripheral tissues. Spiral CT simulation colonoscopy technology can be used in the observation of the obstruction in the proximal intestinal lumen at the time of the complete obstruction of colon cancer [14, 15]. Multislice spiral CT (MSCT) is equipped with wide field detector, so multislice data and images can be obtained simultaneously by one scan. As a result, the resolution of *Z* axis and temporal resolution are greatly enhanced. The enhancement provides more comprehensive and visual tutorial information for the stipulation of preoperative staging plan and treatment plan of stomach cancer and colon cancer, and the incision of stomach cancer and colon cancer under laparoscope [16]. Since multiple factors affect CT images, such as noise and some details being photographed, which make fine particles block the details of images, physicians misdiagnose patients' diseases. Artificial intelligence algorithm can address the problems in CT images, such as noise, and avoid image details being blocked by fine particles. The application of artificial intelligence algorithm in CT images can help diagnose diseases more accurately and avoid human errors [17]. Nonlocal mean (NLM) algorithm enhances images by utilizing the similarities between image blocks and the redundancy of image information, which can help doctors better recognize CT image information. As one of artificial intelligence algorithms, NLM algorithm is widely applied in CT image recognition.

To sum up, perioperative nursing intervention is the constant focus in clinical application. Because routine nursing has limitations in treating patients, more comprehensive and considerate nursing is essential in taking good care of patients. Hence, the purpose of the study was to discuss the application of traditional NLM algorithm-based CT images in the intervention evaluation of the nursing for patients who performed laparoscopy-guided radical surgery of colon cancer, to enhance the overall diagnostic efficiency and to effectively improve patients' curative rate and prognosis. A total of 100 patients who were diagnosed with colon cancer after enteroscopy and experienced laparoscopic radical surgery in hospital were divided into two groups (one was observation group with comprehensive nursing in operation room and another was control group with routine nursing), each of which included 50 cases. Besides, they all received CT examination. Meanwhile, the improved traditional NLM (INLM) algorithm was proposed, and the effect of image reconstruction by INLM was analyzed. The positive effects of comprehensive nursing in operation room on laparoscopy-guided radical surgery of colon cancer for patients were assessed comprehensively based on the above experiment.

## 2. Materials and Methods

### 2.1. Research Objects

A total of 100 patients who were diagnosed with colon cancer after enteroscopy and experienced laparoscopic radical surgery in hospital between December 2018 and March 2021 were chosen as the research objects. These patients ranged from 25 to 65 years old. They were divided equally into an observation group and a control group. This research had been approved by the ethics committee of the hospital, and patients as well as their family members were informed about the research. Patients signed an informed consent form.

Inclusion standards are as follows: (a) patients who suffered from disease within three months; (b) patients willing to engage in the research and sign the informed consent form; (c) patients not being nursed; (d) patients older than 18 years old; (e) patients whose clinical symptoms were mainly abdominal distention and stomachache.

Exclusion standards are as follows: (a) patients with mental diseases; (b) patients with other malignant tumors; (c) patients who quitted during the experiment.

### 2.2. Nursing Method

Routine nursing intervention was adopted in the control group. Routine fasting and ban on drinking before surgery; catheter encumbrance; routine examination; and examination of patients' body temperature, blood pressure, and heart rate were performed to monitor the change of disease.

Comprehensive nursing intervention in operation room was adopted in the observation group. (a) Patients were asked if they have ever been allergic, smoked, or suffered from any chronic diseases, such as coronary heart disease and high blood pressure. Besides, auxiliary examination of patients was performed, and then targeted nursing plan was made based on patients' individual disease. (b) Nursing staff needed to observe patients' mentality and provide psychological counseling for patients to receive surgery calmly. (c) Patients were instructed to keep a comfortable posture during surgery to enable nursing staff to establish intravenous access. Meanwhile, the index levels of each vital sign of patients were closely monitored, such as heart rate, blood pressure, and pulse. During surgery, nursing staff should work cooperatively with doctors in intraoperative care. (d) Postoperative visit was essential. Nursing staff should help patients remove pillows, lie on their backs, and tilt their heads slightly to one side to avoid aspiration by mistake before patients woke. Meanwhile, electrocardiogram monitoring should be strengthened. When patients woke, they should be asked to keep semi-reclining position to make patients undergo peritoneal drainage by spontaneous ventilator. In addition, patients should be reminded that they need to get out of bed to do physical exercise as early as possible to promote the recovery of intestinal function and to avoid deep vein thrombosis of lower limbs.

Observation indexes are as follows: (a) the operation time and the amount of intraoperative blood loss; (b) the time of getting out of bed, ventilation recovery time, postoperative meal time, stomach tube encumbrance time, and catheter encumbrance time.

### 2.3. CT Examination

LightSpeed 64-row spiral CT instrument was adopted. Before scan, patients were forbidden to eat for 12 hours. Patients were asked to take 800 mL of liquor containing 30 mL of contrast agent mixed with warmed boiled water 72 hours before scanning. The scan ranged from the top of the diaphragm to the lower margin of the symphysis pubis, including the whole colon and rectum. The scan parameters are as follows: horizontal scan layer thickness was 10 mm, interlayer spacing was 10 mm, tube voltage was 120 kV, and tube current was 120 mA. About 85 mL of iohexol (injection speed was 3.5 mL/s) was injected into antecubital vein to enhance scan. The thickness of antecubital vein was 1.3 mm, reconstruction increment was 0.8 mm, tube voltage was 120 kV, and tube current was 260 mA.

### 2.4. Improvement of the Nonlocal Mean (NLM) Algorithm

Images are enhanced by traditional NLM algorithm [18] using the similarity between image blocks and the redundancy of image information. In NLM, the pixel value at the point where the pixel point is to be restored is the weight mean of all pixel values in images. If a noise image is *P* and Ω is the pixel set, two equations will be obtained as follows:

(1) $$\begin{array}{c} P=\left\{P\left(i\right)|i\in \Omega \right\}. \end{array}$$

(2) $$\begin{array}{c} P\left(i\right)=\sum_{j\in H}s\left(i,j\right)P\left(j\right). \end{array}$$

In equations (1) and (2), *H* represents search window, *s*(*i*, *j*) refers to the weight of *P*(*j*) and 0 ≤ *s*(*i*, *j*) ≤ 1, ∑_*j*_*s*(*i*, *j*)=1, and *P*(*i*) stands for the filtered pixel value at the point *I* in pixel set. After that, a field is defined to measure the similarity between the pixel points *i* and *j*. The new field is expressed by the following equation:

(3) $$\begin{array}{c} H={\left\{H_{i}\right\}}_{i\in P}. \end{array}$$

In (3), *H*_*i*_ means the similar window centered at the pixel point *i*. The degree of similarity between *i* and *j* is measured by Gaussian weighted Euclidean distance, which is expressed by the following equation:

(4) $$\begin{array}{c} l={\left\|P\left(H_{i}\right)-P\left(H_{j}\right)\right\|}_{2,\lambda }^{2}. \end{array}$$

In (4), *l* refers to the domain Euclidean distance of the pixel point *I* and *λ* represents the standard deviation of Gaussian kernel. In most image blocks, a shorter spatial distance means more similar structure. Therefore, the weight becomes higher when it approaches the targeted pixel. The weight can be expressed by equations (5) and (6) as follows:

(5) $$\begin{array}{c} s\left(i,j\right)=\frac{\sum_{j}e^{\left({\left\|P\left(H_{i}\right)-P\left(H_{j}\right)\right\|}_{2,\lambda }^{2}/f^{2}\right)}}{R\left(i\right)}, \end{array}$$

(6) $$\begin{array}{c} R\left(i\right)=\sum_{j\in H}\exp\left[-\frac{{\left\|P\left(H_{i}\right)-P\left(H_{j}\right)\right\|}_{2,\lambda }^{2}}{f^{2}}\right]. \end{array}$$

In (5) and (6), *R*(*i*) stands for normalization factor and *f* refers to filtering parameter. Nonetheless, this type of weighted calculation paid little attention to the central pixel of image blocks, and it often neglects the difference between gray values in neighborhood of pixel points, which leads to the vagueness of edges. Hence, images are preprocessed by Gaussian filter, a Gaussian template is defined with the size of (2*T*+1)^*∗*^(2*T*+1), and the element size of Gaussian template is shown in the following equation:

(7) $$\begin{array}{c} D_{\lambda }=\frac{\exp\left[-\left(\left(x^{2}+y^{2}\right)/2\lambda^{2}\right)\right]}{2\pi \lambda^{2}}. \end{array}$$

In (7), the values of *x* and *y* are both [−*T*, *L* … 0, *L* … *T*]. Equations (8) and (9) can be worked out by normalization as follows:

(8) $$\begin{array}{c} \text{Nor}D=\sum_{x=-T}^{T}\sum_{y=-T}^{T}D_{\lambda }, \end{array}$$

(9) $$\begin{array}{c} \text{GF}D_{\lambda }=\frac{D_{\lambda }}{\text{Nor}D}. \end{array}$$

In (8) and (9), GF*D*_*λ*_ stands for Gaussian filter and Nor*D* means the result of normalization. Every image block *P*_*i*_ can be expressed by Gaussian filter as follows:

(10) $$\begin{array}{c} C_{D}\left(P_{i}\right)=\text{GF}D_{\lambda }\left(i\right){}^{*}C\left(P_{i}\right). \end{array}$$

In (10), *C*_*D*_(*P*_*i*_) represents the result of Gaussian filter. Weight coefficient is optimized by the mean gradient values of two-pixel neighborhoods. If the horizontal gradient of pixel *i* field is $\bar{w_{x}}$ and the vertical gradient is $\bar{w_{y}}$, the average gradient of pixel field can be expressed by the following equation:

(11) $$\begin{array}{c} \bar{\Delta w}\left(i\right)=\left(\bar{w_{x}},\bar{w_{y}}\right). \end{array}$$

The calculation equation of the gradient angle of pixel points *i* and *j* is shown as follows:

(12) $$\begin{array}{c} \varphi \left(i,j\right)=R\left(\bar{w_{x}},\bar{w_{y}}\right). \end{array}$$

In (12), *φ* stands for the gradient angle. If the angle is larger, the difference between the gradient directions of two neighborhoods becomes more obvious and the degree of similarity is lower. Therefore, exponential decay function is used to improve the effect of the difference between the gradient directions of two neighborhoods on weight. Eventually, the improved weight function is expressed by equations (13)–(16).

(13) $$\begin{array}{c} C_{\text{INLM}}\left(i\right)=Q_{D}\left(i,j\right)C_{D}\left(j\right), \end{array}$$

(14) $$\begin{array}{c} Q_{D}\left(i,j\right)=\frac{e^{\left(-\left({\left\|P_{D}\left(H_{i}\right)-P_{D}\left(H_{j}\right)\right\|}_{2,\lambda }^{2}/f_{1}^{2}\right)-\left({\left\|H_{i}-H_{j}\right\|}_{2,\lambda }^{2}/f_{2}^{2}\right)-\left(\varphi \left(i,j\right)/\beta \right)\right)}}{R\left(i\right)}, \end{array}$$

(15) $$\begin{array}{c} R\left(i\right)=\sum_{j}\exp\left[-\frac{{\left\|P_{D}\left(H_{i}\right)-P_{D}\left(H_{j}\right)\right\|}_{2,\lambda }^{2}}{f_{1}^{2}}-\frac{{\left\|H_{i}-H_{j}\right\|}_{2,\lambda }^{2}}{f_{2}^{2}}-\frac{\varphi \left(i,j\right)}{\beta }\right], \end{array}$$

(16) $$\begin{array}{c} f_{1}{}^{2}=\alpha *\text{mean}{\left\|P_{D}\left(H_{i}\right)-P_{D}\left(H_{j}\right)\right\|}_{2,\lambda }+\kappa . \end{array}$$

**Figure 1:**
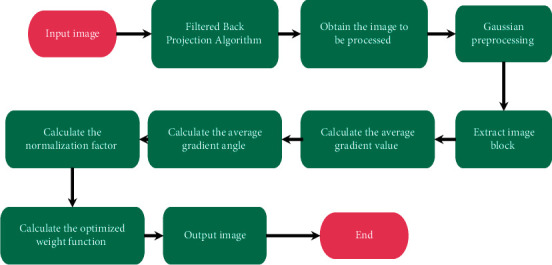
Operational process of the INLM algorithm.

In the above four equations, *C*_*D*_(*j*) means the images preprocessed by Gaussian filter, *Q*_*D*_ refers to weight function optimized in the research, *β* refers to the filtering function that controls the difference between the gradient directions of neighborhoods, *f*_1_ represents the decay factor that controls spatial distance, *f*_2_ stands for decay factor of gray spatial distance, *α* refers to the processing intensity parameter of adjusting filter, and *κ* is used to guarantee that the flat regions of images can obtain sufficient noise reduction strength.  Figure 1 demonstrates the specific procedure of the improvement of NLM by INLM.

### 2.5. Evaluation Indexes of Denoising Effects

Filtered back projection (FBP) [19] and NLM filtering algorithm are introduced and compared with INLM algorithm in the research.

Structural similarity index measure (SSIM) and figure of merit (FOM) are adopted as the evaluation indexes of algorithm denoising effects [20]. SSIM can measure the degree of similarity between two images. A higher degree of the similarity means more obvious relevance between two images. If *E*^*∗*^ represents noise image and *E* refers to denoised image, four equations will be generated as follows:

(17) $$\begin{array}{c} \text{SSIM}\left(E{}^{*},E\right)=a\left(E{}^{*},E\right)b\left(E{}^{*},E\right)c\left(E{}^{*},E\right),\\ a\left(E{}^{*},E\right)=\frac{2\eta_{E{}^{*}}\eta_{E}+L_{1}}{2\eta_{E{}^{*}}^{2}\eta_{E}^{2}+L_{1}},\\ b\left(E{}^{*},E\right)=\frac{2\phi_{E{}^{*}}\phi_{E}+L_{2}}{2\phi_{E{}^{*}}^{2}\phi_{E}^{2}+L_{2}},\\ c\left(E{}^{*},E\right)=\frac{\phi_{E{}^{*},E}+L_{3}}{\phi_{E{}^{*}}\phi_{E}+L_{3}}. \end{array}$$

In the above equations, *a*(*E*^*∗*^, *E*) means brightness similarity function of two images; *b*(*E*^*∗*^, *E*) refers to contrast similarity function; *c*(*E*^*∗*^, *E*) represents structural similarity function; *η*_*E∗*_ and *η*_*E*_ stand for the averages of noise images and denoised images; *ϕ*_*E∗*_ and *ϕ*_*E*_ refer to the square deviations of noise images and denoised images; and *L*_1_, *L*_2_, and *L*_3_ are all constants.

FOM is used to measure the quality of image edge, whose range is [0,1]. A higher value means high consistency between the edge of actual images and that of ideal images, which is shown in the following equation:

(18) $$\begin{array}{c} \text{FoM}\left(E{}^{*},E\right)=\frac{\sum_{i=1}^{M_{E}}1/\left(1+dr_{i}^{2}\right)}{max\left(M_{E{}^{*}},M_{E}\right)}. \end{array}$$

In (18), *M*_*E*_ refers to the ideal number of pixel points on image edge, *M*_*E*^*∗*^_ means actual number of pixel points on image edge, *d* represents constant, and *r*_*i*_ stands for the distance between the pixel points on image edge and the proximal ideal pixel points on image edge.

### 2.6. Statistical Methods

Statistical Product and Service Solutions (SPSS) 19.0 software was used for research data processing and analysis. Measurement data was denoted by mean ± standard deviation ($\bar{x}$ ± *s*), and enumeration data was expressed by percentage mark (%). Pairwise comparison was made by analysis of variance. *P* < 0.05 indicated that the differences had statistical significance.

## 3. Results

### 3.1. Imaging Performance of the INLM Algorithm

**Figure 2:**
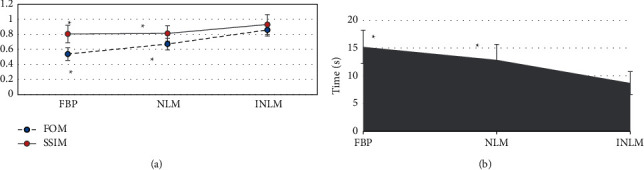
Analysis of the imaging performance of the INLM algorithm: (a) FOM and SSIM; (b) the average running time. *∗* means that the differences had statistical significance compared with the INLM algorithm (*P* < 0.05).

Figure 2 shows that SSIM and FOM index of INLM algorithm were both obviously higher than those of FBP algorithm and NLM algorithm, while the average running time of INLM algorithm was significantly less than that of FBP algorithm and NLM algorithm. There was statistical significance of the differences (*P* < 0.05).

**Figure 3:**
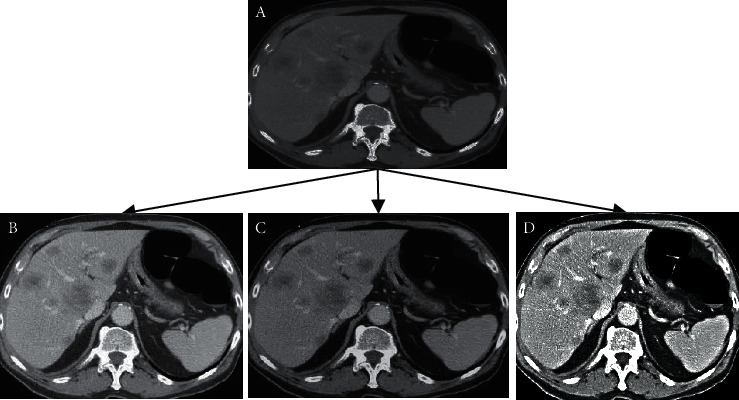
Comparison of the reconstructed images of INLM, FBP, and NLM algorithms: A refers to the original image; B, C, and D stand for the reconstructed images of FBP, NLM, and INLM algorithms, respectively.

Figure 3 demonstrates that the images obtained after the reconstruction of three algorithms were obviously improved compared with original images, including the reduction of artifact and noise and the enhancement of the definition of tissues. Among three reconstructed algorithms, the overall visual effects of reconstructed images of INLM algorithm were more significant than those of the images of other two algorithms, including the improvement of definition and noise.

### 3.2. Comparison of Basic Information about Patients in Two Groups

**Figure 4:**
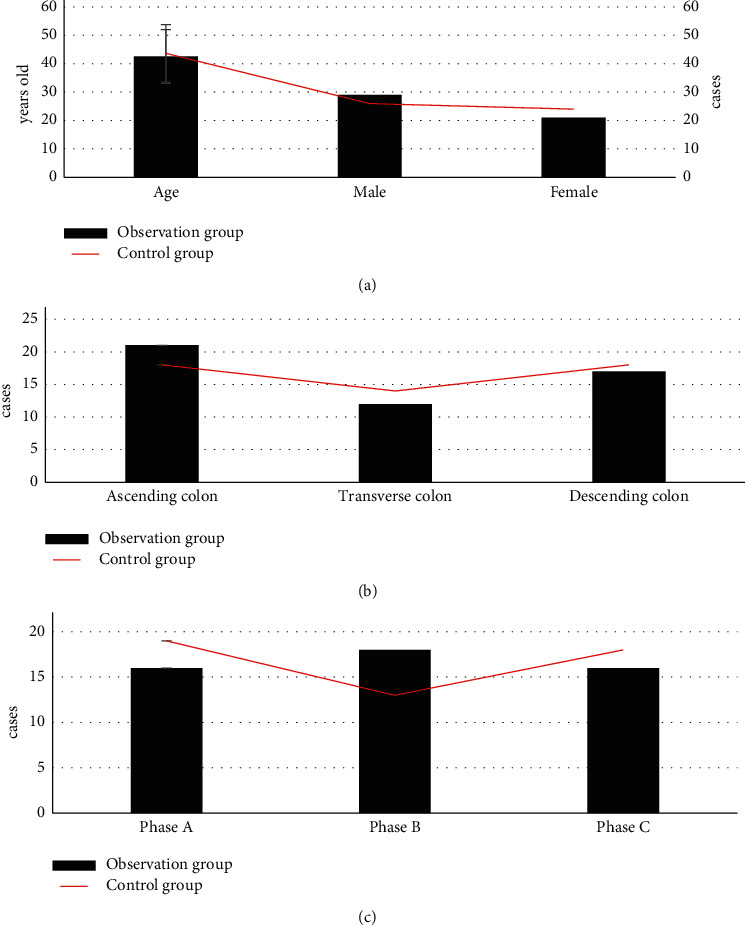
Comparison between basic information of patients in two groups: (a) age and gender ratio; (b) pathological types (ascending colon, transverse colon, and descending colon); (c) Dukes staging (phase A, phase B, and phase C).

Figure 4 presents age, gender ratio, pathological types (ascending colon, transverse colon, and descending colon), and Dukes staging (phase A, phase B, and phase C). All pairwise comparisons had no statistical significance (*P* > 0.05).

### 3.3. Imaging Data of Some Cases

**Figure 5:**
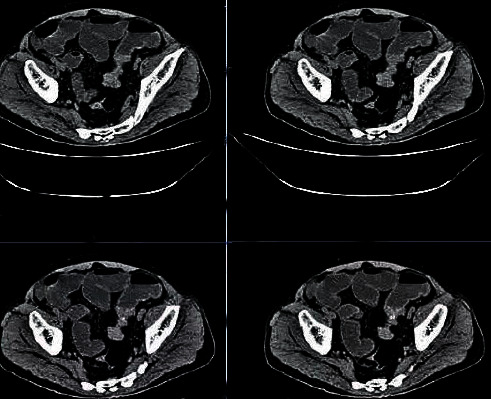
CT image of a 52-year-old male patient complaining mainly about loose stools, changes in excrement, and occasional stomachache for more than one year.

Figure 5 demonstrates that intestinal walls of patients with colon cancer were obviously thickened in CT images. Irregular localized or diffusive thickening occurred commonly on intestinal walls of colon cancer patients. Lesions were eccentric with irregular forms. CT enhancement scan indicated that homogenous enhancement was obvious.

**Figure 6:**
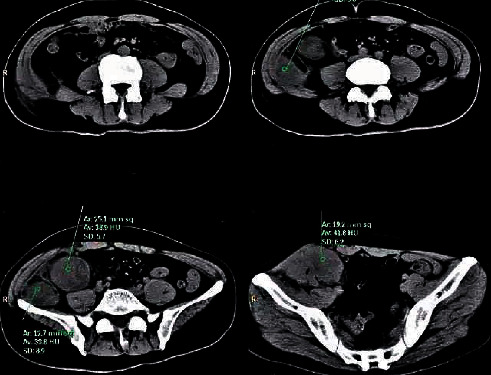
CT image of a 50-year-old male patient complaining about repeated right and lower abdominal pain with abdominal mass for over two months.

Figure 6 shows that intestinal walls were thickened irregularly and lumps were formed. There was certain degree of enhancement in tumors. The boundary between tumors and adjacent structures was vague, which implied that adjacent structures were invaded.

**Figure 7:**
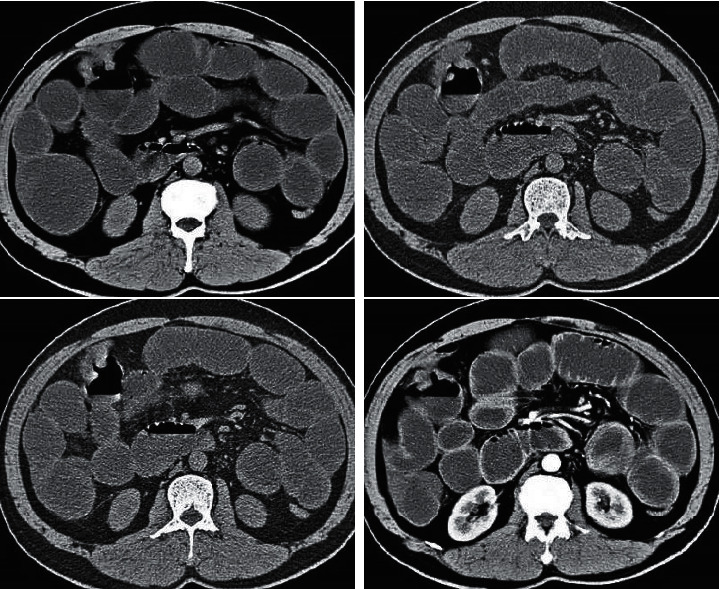
CT image of a female patient at the age of 55. She complained mainly about stomachache and abdominal distention, which lasted for over one month. Besides, there was no obvious incentive more than one month before admission to hospital. However, she suffered from discontinuous abdominal distention and peripheral umbilicus paroxysmal colic along with nausea and emesis. Vomitus was excreted from stomach. After emesis, abdominal pain was alleviated to some extent along with belching and heartburn.

Figure 7 demonstrates that there were irregular soft tissue mass shadows in curved enteric cavity of colonic liver, and they were obviously enhanced. Besides, local tube walls were thickened, lumens were narrow, serosal surface was crude, and gap density among peripheral fat was slightly widened. There were no obvious lymph glands. However, ascending colon and small intestine diffused and expanded. In addition, there were effusion and pneumatosis inside them. Gas and liquid equilibrium were observed.

### 3.4. Comparison of Operation Time and Amount of Intraoperative Blood Loss between Patients in the Observation Group and Control Group

**Figure 8:**
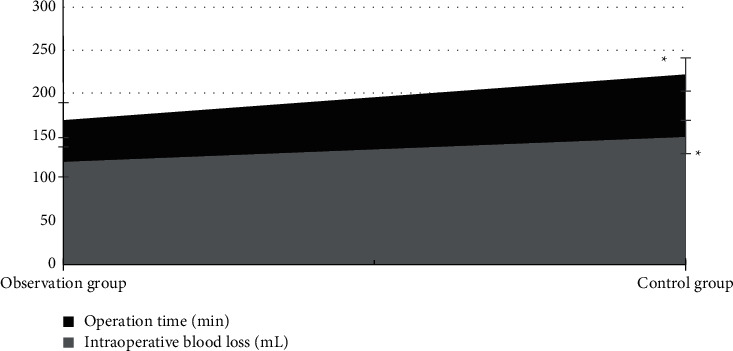
Comparison of operation time and amount of intraoperative blood loss between patients in the observation group and control group. Note: ^*∗*^ means the differences between the observation group and control group had statistical significance (*P* < 0.05).

According to Figure 8, the operation time and amount of intraoperative blood loss of patients in the observation group were both obviously less than those of patients in the control group. The difference had statistical significance (*P* < 0.05).

### 3.5. Comparison of Postoperative Indexes of Patients in the Observation Group and Control Group

**Figure 9:**
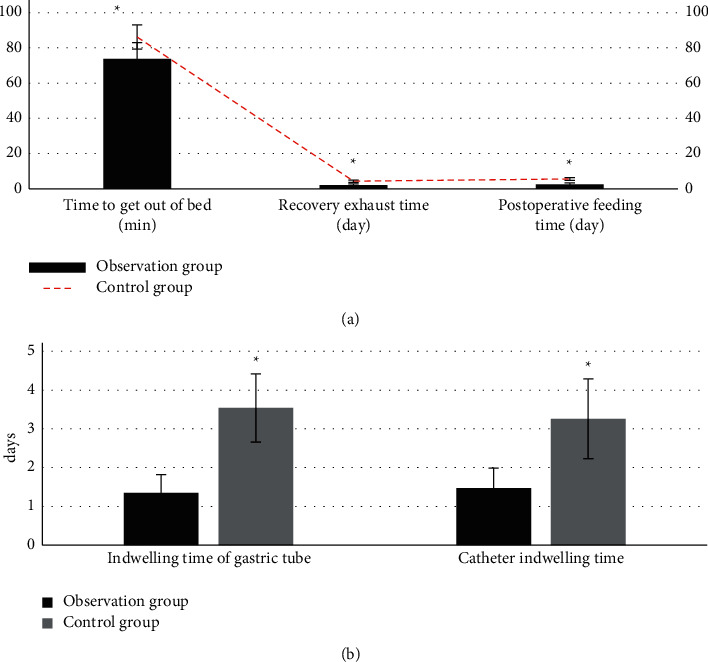
Comparison of postoperative rehabilitation indexes of patients in the two groups: (a) time of getting out of bed, ventilation recovery time, and postoperative meal time; (b) stomach encumbrance time and catheter encumbrance time. ^*∗*^ shows that the differences between the control group and observation group had statistical significance (*P* < 0.05).

As Figure 9 shows, time to get out of bed, recovery exhaust time, postoperative feeding time, indwelling time of gastric tube, and catheter indwelling time of patients in the observation group were all less than those of patients in the control group. The differences had statistical significance (*P* < 0.05).

### 3.6. Comparison of Complications of Patients in the Observation Group and Control Group

**Figure 10:**
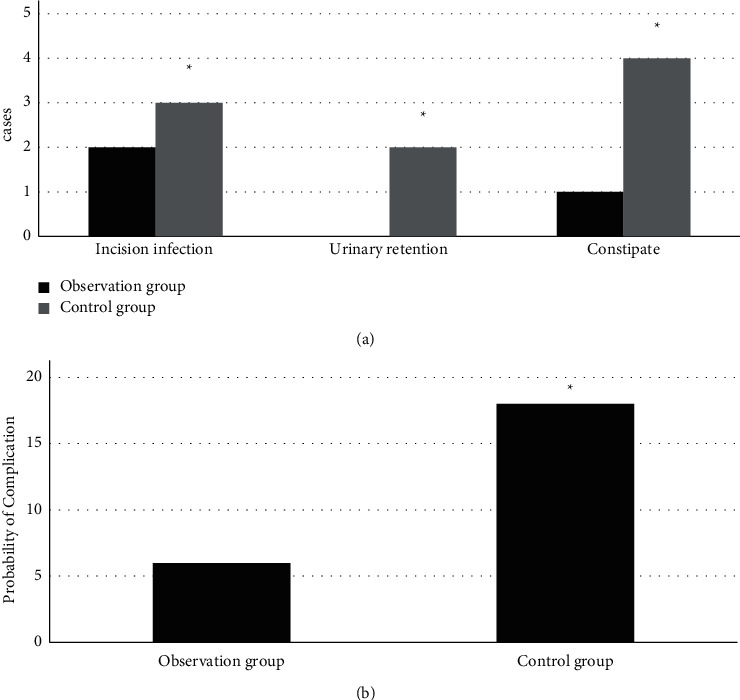
Comparison of complications of patients in the observation group and control group: (a) the postoperative simultaneous incision infection, urinary retention, and number of cases with constipation; (b) the incidence of postoperative complications. ^*∗*^ means that the differences between the observation group and control group had statistical significance (*P* < 0.05).

According to Figure 10, there were 3 cases with postoperative complications in the observation group, and 2 out of them got incision infection while 1 suffered from constipation. In the control group, there were 9 cases with postoperative complications. Among these cases, 3 had incision infection, 2 suffered from urinary retention, and 4 got constipation. The comparison of the incidence of complications revealed that the incidence of postoperative complications (6%) of patients in the observation group was significantly lower than that of patients in the control group (18%), and the differences had statistical significance (*P* < 0.05).

## 4. Discussion

In general, surgical treatment is adopted in the treatment of colon cancer, but conventional open surgery is very traumatic and causes great pains to patients. At present, as medical technology develops, laparoscopy-guided radical surgery of colon cancer is gradually applied to replace open surgery because it is minimally invasive and causes little pain, and patients can recover in a short time. Besides, considerate and comprehensive nursing is required for the adoption of this technology. Based on routine nursing, comprehensive nursing in operation room is helpful for psychological intervention and rehabilitation guidance for patients and can meet the current clinical nursing needs [21, 22]. Hence, a total of 100 patients who were diagnosed with colon cancer after enteroscopy examination and received laparoscopic radical surgery were chosen as the research objects, and they were equally divided into an observation group (comprehensive nursing) and a control group (routine nursing). All patients in the two groups received CT examination. The comparison of basic information of these patients indicated that the differences of patients in the two groups in terms of age, gender ratio, pathological types (ascending colon, transverse colon, and descending colon), and Dukes staging (phase A, phase B, and phase C) had no statistical significance (*P* > 0.05). Because of the results, subsequent research is feasible. To improve the quality of CT images, the improved traditional NLM algorithm (INLM) was proposed, and it was analyzed and compared with FBP algorithm and NLM algorithm. The results demonstrated that SSIM and FOM of INLM were both obviously higher than those of FBP algorithm and NLM algorithm, while the average running time of INLM algorithm was less than that of FBP and NLM algorithm. The differences between the indexes of three algorithms had statistical significance (*P* < 0.05), which was similar to the results of the analysis of image reconstruction algorithm conducted by Lee et al. [23]. The similarity of these results demonstrated that the proposed INLM algorithm could improve the accuracy of image reconstruction by traditional algorithm, shorten running time, and enhance overall diagnostic efficiency.

To further analyze the effects of comprehensive nursing intervention in operation room on patients who received laparoscopy-guided radical surgery, the operation time and amount of intraoperative blood loss of patients in the two groups were recorded. The results showed that the operation time and amount of intraoperative blood loss of patients in the observation group were both less than those of patients in the control group, and the differences had statistical significance (*P* < 0.05). The outcome of the comparison demonstrated that comprehensive nursing in operation room could improve the surgical effects of laparoscopic radical surgery of colon cancer and mitigate the trauma to patients [24]. Moreover, the time of getting out of bed, ventilation recovery time, postoperative meal time, stomach encumbrance time, and catheter encumbrance time of patients in the observation group were all less than those of patients in the control group, and the differences had statistical significance (*P* < 0.05). These differences further proved that comprehensive nursing in operation room using laparoscopic radical surgery of colon cancer could enhance the curative rate and promote the recovery of patients effectively. Therefore, this medical technology was worthy of clinical promotion and adoption. The comparison of postoperative complications showed that there were 3 cases with complications in the observation group: 2 with incision infection and 1 with constipation. In the control group, there were 9 cases suffering from postoperative complications: 3 with incision infection, 2 with urinary retention, and 4 with constipation. The results indicated that comprehensive nursing in operation room played a significant role in the prognosis of patients who received laparoscopic radical surgery of colon cancer because patients receiving comprehensive nursing in operation room recovered better than those who received routine nursing.

## 5. Conclusion

The result showed that the INLM proposed in this research could enhance the accuracy of image reconstruction by traditional algorithm and shorten running time as well as improving overall diagnostic efficiency. Comprehensive nursing in laparoscopic radical surgery of colon cancer could improve the curative rate and prognosis of patients effectively. Therefore, this technology is worthy of promotion and adoption. However, the samples of INLM were not tested on a large scale, so whether this algorithm can be applied directly in clinical CT images is an issue to be further discussed. Besides, the small sample size of patients may result in bias. As a result, data of patients should be collected in further research, and artificial intelligence (AI) algorithm as well as imaging technology should be applied in clinical analysis. To sum up, the research provides statistical references for the nursing intervention measures for patients receiving laparoscopy-guided radical surgery of colon cancer.

## Data Availability

The data used to support the findings of this study are available from the corresponding author upon request.
